# cERBB-2/Her-2 Neu Overexpression and Prognostic Significance in Uterine Carcinosarcoma*

**DOI:** 10.5146/tjpath.2022.01588

**Published:** 2023-01-15

**Authors:** Huseyin Salih Semiz, Emel Ebru Pala, Behzat Can, Elif Atag, Hatice Gungor, Muzaffer Sancı

**Affiliations:** Department of Medical Oncology, Dokuz Eylül University Institute of Oncology, Izmir, Turkey; Department of Pathology, Health Science University, Tepecik Education and Research Hospital, Izmir, Turkey; Department of Gynecological Oncology, Health Science University, Tepecik Education and Research Hospital, Izmir, Turkey; Department of Medical Oncology, Health Science University, Istanbul Haydarpaşa Numune Education and Research Hospital, Istanbul, Turkey

**Keywords:** Carcinosarcoma, Her-2/neu, Prognosis, Uterine cancer

## Abstract

***Objective:*** There is not enough data in the literature regarding Her-2 overexpression in uterine carcinosarcomas or its association with the prognosis. The aim of this study was to determine the Her-2 overexpression rate in uterine carcinosarcoma and to evaluate its relationship with the prognosis.

***Material and Method:*** Her-2 protein and gene status were evaluated by immunohistochemistry (IHC) and fluorescence in situ hybridization (FISH), respectively, in hysterectomy specimens from 51 patients with uterine carcinosarcoma.

***Results:*** Her-2 protein expression in the epithelial component was negative in 42 patients (score 0 in 33 cases, score (+1) in 9 cases), score (+2) in 7 patients and score (+3) in 2 patients. None of the patients had Her-2 protein expression within the sarcomatous component of the tumors. Her-2 gene was not amplified in epithelial or mesenchymal tumor areas according to the FISH method. There was no difference between the Her-2 overexpression negative and positive groups in terms of disease-free survival (DFS) and overall survival (OS). Her-2 overexpression was significantly higher in tumors of patients diagnosed at 65 years or older (p=0.046).

***Conclusion:*** In our study, no relationship could be shown between Her-2 overexpression and prognosis in uterine carcinosarcoma. More comprehensive studies are needed to illustrate the relationship between Her-2 overexpression and carcinosarcoma prognosis.

## INTRODUCTION

Carcinosarcomas (CS) constitute 5% of all uterine cancers and have a poor prognosis (1). They have both mesenchymal and epithelial components. Histology of the epithelial component can be endometrioid, serous, clear cell, or undifferentiated. The mesenchymal component can be homologous, as in endometrial stromal sarcoma (ESS), leiomyosarcoma (LMS), or undifferentiated sarcoma, or heterologous as in rhabdomyosarcoma, osteosarcoma, chondrosarcoma, liposarcoma, or fibrosarcoma (2).

Carcinosarcomas are considered to develop from an epithelial origin. It is known that the epithelial and mesenchymal components of carcinosarcomas have many common features, both immunohistochemically and genomically. In addition, carcinosarcomas have similar characteristics to uterine carcinomas rather than uterine sarcomas in terms of the clinical course and risk factors (3). Approximately 70% of patients are at locally advanced or metastatic stage at the time of diagnosis (4).

The most important prognostic factor in carcinosarcomas is the stage of the disease. While 5-year survival is around 60% in stage 1 disease, this rate decreases to below 10% in stage 4 (5). Other prognostic factors are depth of myometrial invasion, presence of lymphovascular invasion, advanced age, ethnicity (poor prognosis in black race), and late menopause (6). The poor prognosis of the disease and the lack of treatment options other than conventional chemotherapies, especially in advanced stage disease, have led to the search for new biomarkers and treatments.

ERBB-2 (Her-2 / neu) is a member of the epidermal growth factor receptor (EGFR) family. In many types of cancer, especially in breast carcinoma, overexpression of Her-2 is associated with poor prognosis, and it is also associated with poor prognosis in endometrial serous carcinoma (7–9). However, there is not enough data in the literature regarding Her-2 overexpression in uterine carcinosarcomas or its association with the prognosis. In this study, we aimed to determine Her-2 overexpression/amplification rates in uterine carcinosarcomas and its relationship with the prognosis.

## MATERIAL and METHODS

Sixty consecutive uterine carcinosarcoma cases diagnosed in our center between 2013 and 2020 provided consent and were selected for the study. Paraffin blocks with samples for nine patients were insufficient for examination and therefore excluded from the study. Samples for a total of fifty-one patients were examined. Since our study was cross-sectional and carcinosarcoma cases are seen much less than other uterine malignancies, the sample size was not calculated. All cases in the specified date range were included in the study.

Her-2 overexpression was determined by immunohistochemistry (IHC) and consequently fluorescence in situ hybridization (FISH), and scoring was done as either negative (score 0 and score (+1), score (+2) and score (+3). IHC score (+2) and score (+3) cases were accepted as positive for Her-2 overexpression. The correlation between Her-2 overexpression and survival (DFS and OS) was examined. We also studied the correlation between Her-2 overexpression and prognostic parameters for carcinosarcoma such as necrosis, atypia, FIGO stage, LVI, PNI, and histological grade.

### Her-2 Immunohistochemistry Staining Procedure

Formalin-fixed, paraffin-embedded tissues from hysterectomy specimens were processed according to standardized protocols. Paraffin blocks containing both epithelial and mesenchymal areas were chosen. Her-2 protein expression was evaluated by IHC, and Her-2 gene amplification was evaluated by FISH method. To determine the Her-2 protein expression, polyclonal rabbit Her-2 antibody (1/300 dilution) (A0485 clone, DAKO) was used. The whole procedure was performed on a DAKO Autostainer Link 48. Her-2 staining was evaluated according to the College of American Pathologists (CAP) 2018 protocols for breast cancer Her-2 scoring (10).

### Fluorescence in Situ Hybridization Procedure of Her-2

DAKO instant quality FISH kit was used for determining the Her-2 gene copy numbers. Deparaffinization of slides was started on the incubator at 60 °C for one hour, then run through two xylol series. After rehydration with ethanol, slides were pretreated with pretreatment solution for 10 minutes at 99 °C in a water bath. Ready-to-use pepsin was used for enzymatic digestion for 4 min at 37 °C on the hybridizer. Her-2/CEN17 probe mix was applied (10 µL) to each slide. After denaturation at 66 °C for 10 min, hybridization was performed at 45 °C for 2 hours. At the end of the hybridization, the slides were washed with wash buffer at 63 °C for 10 min. Then 15 µL of fluorescence mounting medium was applied to the dehydrated slides and cover slipped.

### Interpretation of Her-2 FISH method

Epithelial and mesenchymal areas were evaluated with an Olympus BX51 fluorescence microscope equipped with red/green/DAPI filter set under oil immersion objective (100x). CAP 2018 recommendations were used for evaluation of Her-2 gene status (10).

### Statistical Analyses

The data was analyzed with the SPSS 24.0 package program. Numerical data analysis was done with Fisher’s Exact test and the Chi-Square test. The Mann-Whitney U Test was used for independent group analysis. The Kaplan-Meier test was used for survival analysis. Survival curves were compared using Log Rank analysis. Statistical significance level was accepted as p <0.05.

## RESULTS

Fifty-one uterine CS patients diagnosed between January 2013 and December 2020 were enrolled in this study. The median age was 63.4 years (range: 43-81) and only 3 of the patients were premenopausal at the time of diagnosis. While 15 patients had no comorbidity (29.4%), all the remaining patients (70.6%) had at least one comorbid disease (type 2 diabetes, hypertension, etc.). Patient sociodemographic and clinicopathological characteristics are shown in Table 1.

**Table 1:** Clinicopathological characteristics of patients.

**Parameters**		**Number (n)**	**Percentage (%)**
Median age at diagnosis		63 (Range: 43-81)	
Body mass index (median)		30.76 (Range: 23-46)	
Premenopausal		3	5.9
Postmenopausal		48	94.1
Prior tamoxifen use		3	5.9
At least one comorbidity		36	70.6
Surgical Procedure	TAH+BSO	1	2
	TAH+BSO+PPLND	3	5.9
	TAH+BSO+PPLND+omentectomy	44	86.3
	inoperable	3	5.9
FIGO Stage	1A	8	15.7
	1B	12	23.5
	2	7	13.7
	3B	2	3.9
	3C1	7	13.7
	3C2	9	17.6
	4A	1	2.0
	4B	5	9.8
Mesenchymal component	Homologous	39	76.5
	Heterologous	12	23.5
Tumor histology (carcinoma component)	Endometrioid	25	49
	Serous	23	45.1
	Undifferentiated	3	5.9

**TAH:** Total abdominal hysterectomy, **BSO:** Bilateral salghingoopherectomy, **PPLND:** Pelvic-paraaortic lymph node dissection

Twenty-two patients were diagnosed with carcinosarcoma by probe curettage and proceeded to cytoreductive surgery. Operation materials were also found to be compatible with carcinosarcoma. Histopathological findings of probe curettages are shown in Table 2. The histological subtypes of the mesenchymal component in the operation materials are given in Table 3. Thirty-seven patients received carboplatin and paclitaxel, while six patients received ifosfamide and paclitaxel as adjuvant chemotherapy after frontline cytoreductive surgery. Four patients did not receive adjuvant chemotherapy due to a poor performance score (ECOG performance score >2) and three patients for unknown reasons. At the time of diagnosis, 3 of 51 patients were inoperable. Optimal cytoreductive surgery was performed in these 3 patients after neoadjuvant carboplatin and paclitaxel chemotherapy. After adjuvant chemotherapy, 8 patients received brachytherapy, 4 patients received external radiotherapy, and 13 patients received both brachytherapy and external radiotherapy. A total of 22 patients did not receive adjuvant radiotherapy.

**Table 2:** Histopathological characteristics of probe curettage.

**Histologic Type**	**Number (n)**	**Percent (%)**
Carcinosarcoma	22	43.1
Endometrioid Carcinoma	22	43.1
Adenosarcoma	2	3.9
Sarcomatoid Carcinoma	1	2.0
Not available	3	5.9
Missing	1	2.0
**Total**	**51**	**100**

**Table 3:** Histological subtypes of mesenchymal components.

**Histological Subtype**	**Number (n)**	**Percent (%)**
Leiomyosarcoma	2	3.9
Rabdomyosarcoma	5	9.8
Endometrial stromal sarcoma	2	3.9
Undifferentiated sarcoma	27	52.9
Chondrosarcoma	3	5.9
Mixed	7	13.7
Pleomorphic sarcoma	5	9.8
**Total**	**51**	**100.0**

**Figure 1:**
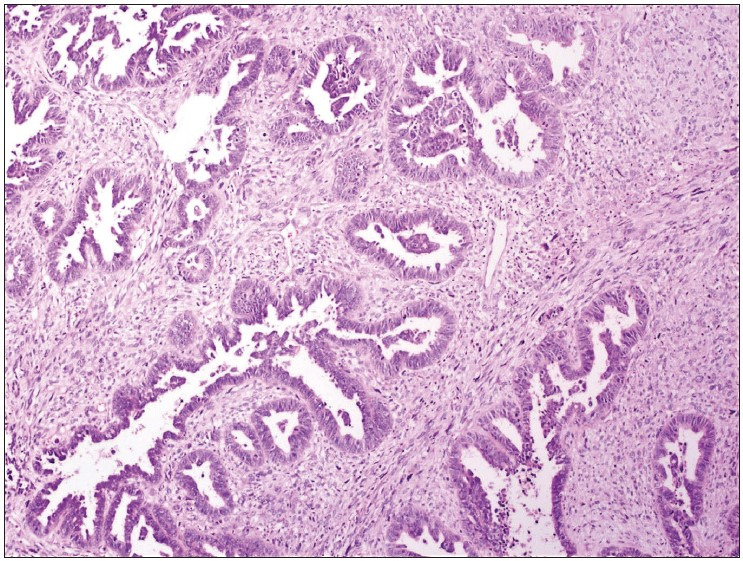
Biphasic tumor areas with high grade epithelial and mesenchymal components (HE, 10x).

**Figure 2:**
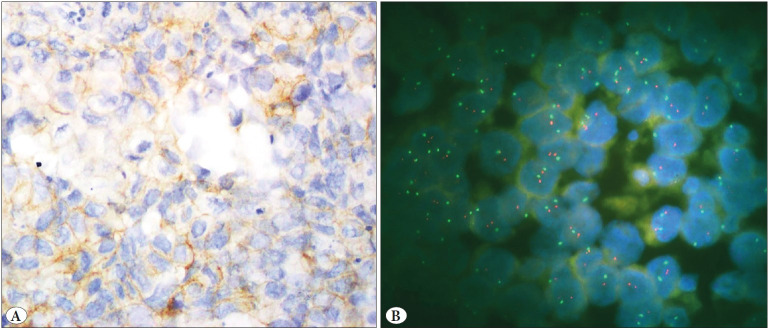
**A)** Carcinosarcoma case showing incomplete membranous staining (1(+) score) in epithelial component by HER2 immunohistochemistry is **B)** non-amplified by HER2 fluorescence in situ hybridization.

**Figure 3:**
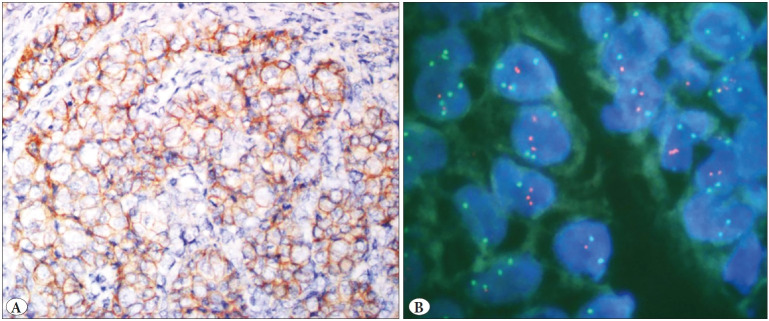
**A)** Carcinosarcoma case showing mild/moderate complete membranous staining (2(+) score) in carcinoma component by HER2 immunohistochemistry is **B)** non-amplified by HER2 fluorescence in situ hybridization.

**Figure 4:**
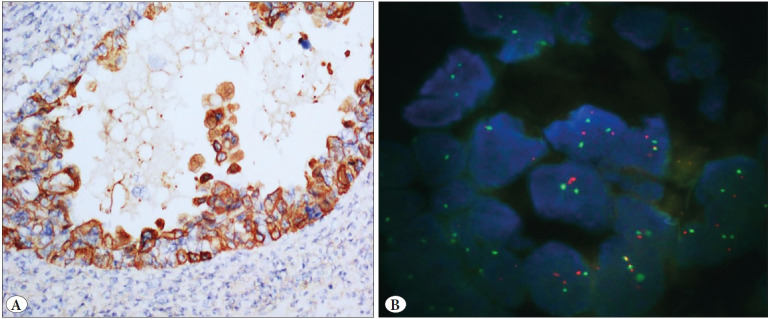
**A)** Carcinosarcoma case showing complete membranous staining (3(+) score) in 20% of epithelial tumor component by HER2 immunohistochemistry is **B)** non-amplified by HER2 fluorescence in situ hybridization.

IHC and FISH were carried out on hysterectomy specimens of 51 patients. Biphasic tumor areas with high-grade epithelial and sarcomatous components (HE, 10x) are shown in Figure 1. Her-2 protein expression in epithelial areas was score (0) in 33, score (+1) in 9, score (+2) in 7, and score (+3) in 2 cases by A0485 IHC. Her-2 expression in mesenchymal areas was negative according to IHC in all cases. None of the cases showed Her-2 gene amplification in epithelial or mesenchymal areas according to the FISH method (Figure 2, Figure 3, Figure 4).

At the time of data cut-off, 31 patients were alive, and 12 patients had no evidence of disease. Median follow-up period was 16 months. Median DFS was 18 months, and median OS was 27 months. There was no significant difference between the histological subtypes of epithelial components in terms of the Her-2 positivity rate (p=0.9) (Table 4).

**Table 4:** Her-2 positivity according to histological subtypes of epithelial components.

**Epithelial Component**	**Her-2 Positivity**				**Total, n (%)**
	**Negative, n (%)**	**(+1), n (%)**	**(+2), n (%)**	**(+3), n (%)**	
Endometrioid	20 (60.6)	2 (22.2)	1 (14.3)	1 (50.0)	24 (47.1)
Serous	10 (30.3)	5 (55.6)	4 (57.1)	1 (50.0)	20 (39.2)
Mixed	2 (6.1)	1 (11.1)	2 (28.6)	0 (0.0)	5 (9.8)
Undifferentiated Carcinoma	1 (3.0)	1 (11.1)	0 (0.0)	0 (0.0)	2 (3.9)

**Figure 5:**
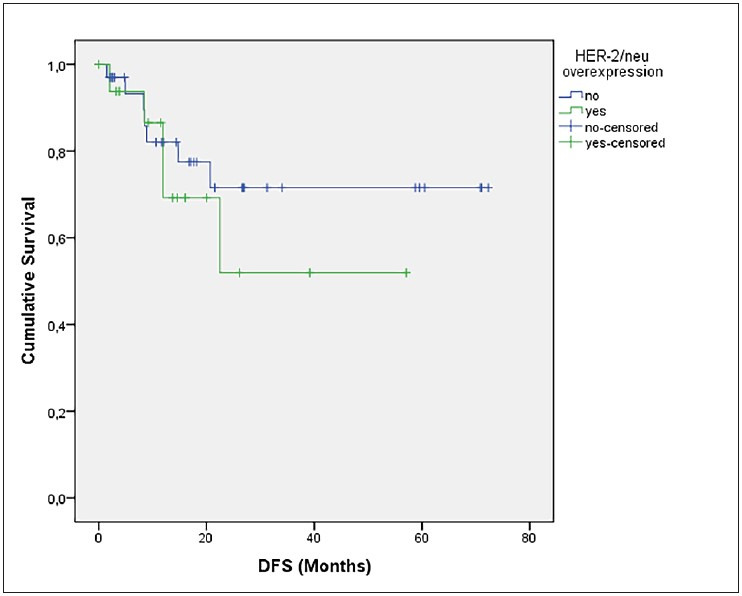
DFS stratified by Her-2 positivity

**Figure 6:**
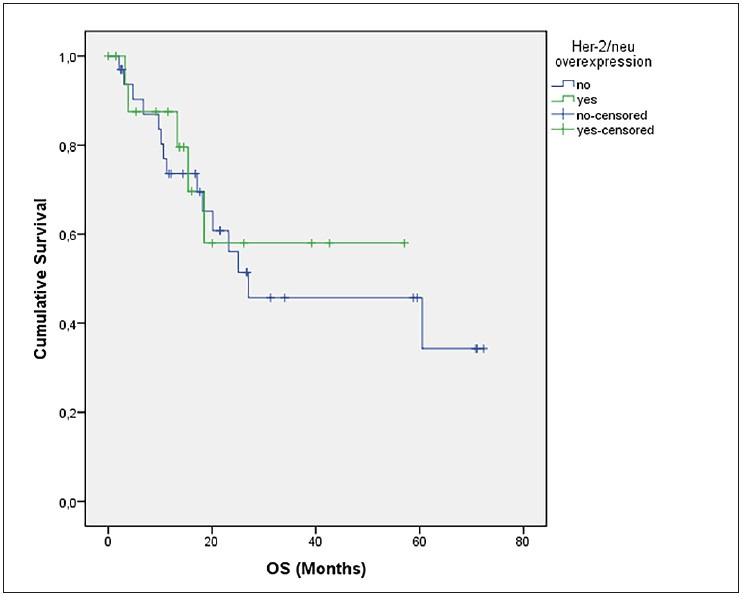
OS stratified by Her-2 positivity

The median age was 69.31 in the Her-2 score (+2) and (+3) groups (n=9) and 62.88 in Her-2 score (0) and (+1) groups (n=42) (p=0.04) The estimated mean DFS did not differ between Her-2 negative and positive groups (p=0.59) (Figure 5). There was no difference between the Her-2 negative and positive groups in terms of overall survival (39.8 vs. 38.3 months, p=0.698) (Figure 6). There was no relationship between Her-2 expression scores and any of the parameters (necrosis, atypia, FIGO stage, LVI, PNI, histological grade) with negative prognostic value for carcinosarcoma. However, the probability of recurrence was higher in patients with cervical involvement than in patients without cervical involvement (p=0.016) (Table 5).

**Table 5:** Crosstabulation of clinical characteristics of uterine carcinosarcoma patients.

	**Her-2 Positivity in Epithelial Component**				
	**No**		**Yes**		p value*=0.038
**Age Group**	**n**	**%**	**n**	**%**	
**<65**	21	77.8	6	22.2	
**≥65**	12	50.0	12	50.0	
	**Relapse**				p value**=0.247
	**No**		**Yes**		
**Age**	62.58 (58.08 – 70.17)		69.04 (60.01 – 72.17)		
	**Status**				p value*=0.247
	**Alive**		**Ex**		
**Her-2 positivity in Epithelial Component**	**n**	**%**	**n**	**%**	
**No**	18	54.5	15	45.5	
**Yes**	13	72.2	5	27.8	
	**Relapse**				p value***=0.036
	**No**		**Yes**		
**Cervical involvement**	**n**	**%**	**n**	**%**	
**No**	26	86.7	4	13.3	
**Yes**	10	55.6	8	44.8	

*Chi-square test, **Mann-Whitney U test, ***Fisher’s exact test

The frequency of Her-2 [score (+2) and (+3)] was significantly higher in patients 65 years of age and older (29.1% positivity) than patients under 65 years of age (7.4% positivity) (p=0.04). Although not statistically significant, the median age in patients with disease recurrence was higher than in patients without recurrence (p=0.247) (62.58 vs. 69.04) (Table 5). Also, the estimated mean DFS (42.2 months vs. 56.5 months, p = 0.037) and OS (29.6 months vs. 50.4 months, p=0.025) was shorter in the patient group aged ≥ 65 compared to the group aged <65.

## DISCUSSION

Carcinosarcomas are tumors with aggressive behavior and the disease has poor prognosis. However, there is no treatment available other than cytoreductive surgery followed by adjuvant systemic chemotherapy and radiotherapy in early-stage disease and a limited number of systemic chemotherapy regimens in advanced stage. Therefore, the search for targetable biomarkers/molecules in carcinosarcomas continues.

Since Her-2 overexpression was shown to play an important role in treatment and prognosis of serous endometrial carcinomas and the survival advantage of anti-Her-2 treatment was established, we wanted to investigate whether this biomarker could also be a game changer for carcinosarcomas (11).

In our study, we did not observe Her-2 protein overexpression in any mesenchymal component from 51 patients. In contrast, we detected score [(+2) and (+3)] Her-2 expression in the epithelial component in 9 (17.6%) of 51 carcinosarcoma samples.

Her-2 overexpression in uterine carcinosarcoma samples was reported in a range between 0% and 65% by IHC (12,13). Her-2 gene amplification according to the FISH method varied from <1% to 20% of cases in different case series (14,15). Saglam et al. investigated Her-2 immunoexpression in 37 uterine carcinosarcoma cases (16). They found Her-2 score (+3) in only one case (1/37), both in epithelial and mesenchymal areas of the tumor, and (+2) expression in three cases (3/37); however, they did not correlate IHC results with any ISH method.

Sawada et al. and Raspollini et al. also reported higher expression rates of 56% and 29.2% respectively in uterine carcinosarcomas (17,18). Both studies considered (+2) and (+3) results as overexpression. Sawada et al. examined 16 uterine carcinosarcoma cases with Her-2 IHC and reported overexpression in 9/16 cases with 8 of them in the epithelial component, and only 1 of them in the mesenchymal component. They also performed FISH in 6 cases; the epithelial component was examined in 4 cases and the mesenchymal component in 1 case, while the remaining 1 case was undetermined as to whether the component was epithelial or mesenchymal. In 4 cases with Her-2 overexpression, only 1 case had low-level Her-2 DNA amplification (Her-2/CEP17 ratio 2.0), and the remaining 5 cases were either not amplified or the Her-2/CEP17 ratio was <2.0. We also considered score [(+2) and (+3)] results as overexpression (17.6%) based on IHC but protein expression could not be correlated with gene amplification by the FISH method. In our series, Her-2 expression was observed only in the epithelial component of the tumors, compatible with the previous studies.

Her-2 overexpression by IHC and gene amplification by FISH were in perfect concordance in (+3) cases both in breast and gastric carcinomas. Equivocal cases with 20–28% amplification rates need to be clarified by molecular methods. Because of the conflicting results about Her-2 status in uterine carcinosarcomas, we analyzed all our cases both with IHC and FISH methods regardless of their scoring. None of the negative, equivocal, or positive cases according to IHC showed Her-2 amplification based on FISH. We did not observe centromere 17 (CEP17) region amplification. In a study by Yoshida et al. on 89 patients diagnosed with uterine carcinosarcoma, Her-2 staining patterns and what should be used as evaluation criteria were emphasized (19). In this study, Her-2 examination was performed by considering tumor samples as a single histological type without distinction as epithelial and mesenchymal. At the same time, Her-2 staining was examined and compared according to both breast cancer criteria (10) and gastric cancer criteria (20) of the ASCO/CAP guideline. In this study, it was concluded that in the evaluation of Her-2 in uterine carcinosarcoma, the lateral/basolateral staining pattern gave more accurate results instead of the complete membranous staining pattern, and therefore it would be more appropriate to perform the evaluation according to gastric cancer criteria rather than breast cancer criteria. In the study of Yoshida et al., the frequency of Her-2 positive staining was found to be higher than our study. In a study conducted by Rottmann et al. on 80 patients diagnosed with ovarian and uterine carcinosarcoma, the most common staining pattern of Her-2 was found to be incomplete-basal or basolateral staining pattern (21). In this study, which was evaluated according to both 2007 and 2013 breast cancer guidelines of ASCO/CAP, the Her-2 positivity rate was found to be 16%. In our study, the rate of (+1) patients was 17.6%, and the rate of (+2 or +3) patients was 17.7%. The studies of Yoshida and Rottmann provide very important information because, according to these studies the Her-2 staining patterns in uterine carcinosarcomas show significant differences from breast cancer.

The reasons for inconsistencies between Her-2 protein products and Her-2 gene amplification can either be due to tissue fixation/processing problems, aggressive antigen retrieval methods, protein overexpression in transcriptional or posttranslational steps, or chromosome 17 centromere amplification (10). Although there were no cases of false negativity with IHC, we noted score (+3) Her-2 expression without amplification in two cases. Possible reasons for the discordance between IHC and FISH could be the usage of polyclonal antibodies, posttranslational modifications, fixation/processing problems, and aggressive antigen retrieval methods.

In a preclinical study conducted by Nicoletti et al. in the carcinosarcoma cell line, the Her-2 expression level was found to be 25%. In addition, they obtained an antitumoral response with trastuzumab and trastuzumab emtansine (T-DM1), which is a combination of chemotherapy (emtansine) with trastuzumab, in the Her-2 expressing cell group (22). In a study by Raspollini et al., 28 carcinosarcoma cases were examined. In this study, the Her-2 expression level was found to be 32%, and it was concluded that the presence of expression was not related to the prognosis, similar to our research (18). In the study conducted by Livasy et al. with 55 carcinosarcoma cases, the Her-2 expression rate was found to be 25% in the epithelial component and 4% in the mesenchymal component. In addition, this study concluded that Her-2 overexpression had no prognostic significance (15).

Age was a significant parameter between Her-2 score [(0)/(+1)] and score [(+2)/ (+3)] results in our study. Although survival rates were found to be worse in patients aged ≥65 years than in patients under 65 years of age, it is not possible to directly correlate these survival differences with Her-2 expression. The Her-2 score [(+2)/ (+3)] IHC results and gene amplification (FISH test) do not seem to have prognostic significance. Therefore, there is a need to carry out more comprehensive studies.

In our study, Her-2 overexpression was examined by both IHC and FISH methods in all cases to verify the accuracy of the results. Her-2 overexpression in mesenchymal components in our study was also consistent with the literature, in that Her-2 overexpression in other studies was found to be very low-level or negative in the mesenchymal component of carcinosarcomas. Our study has some limitations: we only included a low number of patients with short follow-up period, and therefore the median survival time was not reached in all cases.

The literature about Her-2 overexpression in carcinosarcomas has quite conflicting results. This may be related to the fact that carcinosarcomas are much less common than other uterine malignant tumors and contain highly heterogeneous components. For this reason, it is difficult to obtain large case series and ensure a uniform study. More comprehensive studies and Her-2 mutation analysis are needed to clarify the matter further.

## CONCLUSION

In our study, we did not observe Her-2 protein overexpression in any mesenchymal component in the tumors. In contrast, we detected score [(+2)/ (+3)] Her-2 expression in the epithelial component in 17.6% of carcinosarcoma samples. The Her-2 gene was not amplified in both epithelial and mesenchymal tumor areas according to the FISH method. A relationship between Her-2 overexpression and prognosis in uterine carcinosarcoma could not be detected. More comprehensive studies are needed to show the relationship between Her-2 overexpression and carcinosarcoma prognosis.

## Funding

The Turkish Society of Medical Oncology funded this study with project number 458 on the date 26/11/2020.

## Conflict of Interest

I certify that all of my affiliations with or without financial involvement, within the past 5 years and foreseeable future, and any organization or entity with a financial interest in or financial conflict with the subject matter or materials discussed in the manuscript are completely disclosed (e.g., employment, consultancies, honoraria, stock ownership or options, expert testimony, grants or patents received or pending, and royalties).
